# Proteasome inhibitor (MG132) rescues Na_v_1.5 protein content and the cardiac sodium current in dystrophin-deficient mdx^*5cv*^ mice

**DOI:** 10.3389/fphys.2013.00051

**Published:** 2013-03-26

**Authors:** Jean-Sébastien Rougier, Bruno Gavillet, Hugues Abriel

**Affiliations:** Department of Clinical Research, University of BernBern, Switzerland

**Keywords:** sodium channels, dystrophin, proteasome, proteasome inhibitors, MG132, electrophysiology

## Abstract

The cardiac voltage-gated sodium channel, Na_v_1.5, plays a central role in cardiac excitability and impulse propagation and associates with the dystrophin multiprotein complex at the lateral membrane of cardiomyocytes. It was previously shown that Na_v_1.5 protein content and the sodium current (*l*_Na_) were both decreased in cardiomyocytes of dystrophin-deficient mdx^*5cv*^ mice. In this study, wild-type and mdx^*5cv*^ mice were treated for 7 days with the proteasome inhibitor MG132 (10 μg/Kg/24 h) using implanted osmotic mini pumps. MG132 rescued both the total amount of Na_v_1.5 protein and *l*_Na_ but, unlike in previous studies, *de novo* expression of dystrophin was not observed in skeletal or cardiac muscle. This study suggests that the reduced expression of Na_v_1.5 in dystrophin-deficient cells is dependent on proteasomal degradation.

## INTRODUCTION

The cardiac voltage-gated sodium channel, Na_v_1.5, plays a central role in cardiac function as it is responsible for the depolarization of the cardiac action potential and propagation of cardiac electrical impulses (Nerbonne and Kass, 2005). Mutations in the sodium channel gene, *SCN5A*, are found in patients with a variety of cardiac diseases, such as congenital long QT syndrome type 3 and Brugada syndrome (Wang et al., 1995a, b; Antzelevitch, 2001; Moric et al., 2003). Recent studies have associated mutations in *SCN5A* with dilated cardiomyopathy (Mcnair et al., 2004; Hesse et al., 2007; Mann et al., 2012). Many investigators have characterized naturally occurring *SCN5A* mutations, but little is known about the regulation of expression of Na_v_1.5 in cardiac cells. Recent studies that have reported on Na_v_1.5 interacting partners have suggested that Na_v_1.5 may be part of distinct multiprotein complexes that differ between one cellular compartment and another, and that multiprotein complexes may be involved in the regulation of channel activity, cellular localization, and protein degradation (Tan et al., 2003; van Bemmelen et al., 2004; Mohler and Bennett, 2005; Albesa et al., 2011; Petitprez et al., 2011). Given the important role of Na_v_1.5 in cardiac function, alterations of its regulatory mechanisms could be involved in cardiac diseases of unknown etiology, e.g., only 20% of Brugada syndrome cases have been associated with *SCN5A* mutations (Wilde et al., 2002). Na_v_1.5 associates with the dystrophin multiprotein complex (DMC) at the lateral membrane of cardiomyocytes, as well as to the SAP97 protein at the intercalated disk of cardiac cells (Gee et al., 1998; Gavillet et al., 2006; Albesa et al., 2011; Petitprez et al., 2011). Dystrophin is a 427 kDa cytoplasmic protein which forms a complex at the plasma membrane (Im et al., 1996). In muscle cells, the DMC is thought to strengthen the sarcolemma during contraction by providing a link between the extracellular matrix and the cytoskeleton (Barnabei and Metzger, 2012). Mutations in the dystrophin gene result in Duchene and Becker muscular dystrophies (DMD and BMD), as well as X-linked dilated cardiomyopathy (XLDCM; Towbin et al., 1993). Using the dystrophin-deficient mouse model mdx^*5*cv^, we previously demonstrated that the absence of dystrophin in cardiomyocytes led to a ~50% decrease in the total amount of Na_v_1.5 protein, which was associated with a ~30% decrease in the cellular sodium current (*I*_Na_). In addition, conduction velocity recordings revealed atrial and ventricular conduction slowing, consistent with a ~30% reduction of *l*_Na_ (Gavillet et al., 2006). In parallel, we also demonstrated that in the HEK293 cell line the Na_v_1.5 channel is down-regulated consequently to its ubiquitylation via the ubiquitin ligase activity of Nedd4-2 (van Bemmelen et al., 2004; Rougier et al., 2005). Moreover in mouse cardiac tissue the ubiquitylation of Na_v_1.5 channel has also been shown suggesting a key role of the ubiquitin proteasome system in the regulation of Na_v_1.5 channel *in vivo* (van Bemmelen et al., 2004)

The aim of this study was to elucidate the implication of the ubiquitin proteasome system in the regulation of the Na_v_1.5 channel in control and dystrophin-deficient mdx^*5cv*^ mice. Both strains were treated with the proteasome inhibitor MG132 for 7 days to investigate the potential implication of the proteasome in the down-regulation of Na_v_1.5 channel observed in mdx^*5cv*^ mice. MG132 treatment rescued Na_v_1.5 expression and *I*_Na_ in the cardiomyocytes of mdx^*5cv*^ mice to levels similar to that of the control mice. Proteasome inhibition did not restore dystrophin expression in the skeletal or cardiac muscle of mdx^*5cv*^ mice.

## MATERIALS AND METHODS

### ANIMALS

Wild-type (WT) C57BL/6 mice (Janvier, Le Genest St Isle, France), and C57BL/6Ros-5Cv (mdx^*5cv*^) mice (Jackson laboratories, Bar Harbor, Maine) were raised at the department of pharmacology of the University of Lausanne. Male mice aged 12–16 weeks were used in this study. All animal procedures were performed in accordance with Swiss and Cantonal laws.

### MINI PUMPS

Osmotic mini pumps (ALZET model 1007D, Alzet Osmotic Pump Company, Cupertino, USA) were implanted in the anterior back region of the mice. Pumps were filled up with either a MG132 solution or with the vehicle alone (0.9% NaCl), according to the ALZET filling procedure. MG132 (C2211, SIGMA, Buchs, Switzerland) was delivered at a dose of 10 μg/Kg/24 h. Two millimolars MG132 aliquot were added to dimethylsulfoxide (Merck, Damstadt, Germany), before being further diluted to the appropriate concentration in 0.9% NaCl.

### MICE VENTRICULAR MYOCYTE ISOLATION

Seven days after implantation of the osmotic pump, the mice were heparinized with 100 μl of heparin (Liquemin 5000 IU/ml, Roche, Basel, Switzerland). They were then euthanized with an intraperitoneal injection of pentobarbital. The hearts were excised, rinsed in Krebs solution, mounted on a Langendorff apparatus and subjected to collagenase retroperfusion. The procedure for mice ventricular myocyte isolation was previously described in detail (Gavillet et al., 2006). Approximately 10% of the isolated myocytes were plated on a laminin coated dish and used for patch clamp measurements; the remaining myocytes were frozen in pellet form. The frozen pellets were subsequently used for mRNA or protein extraction.

### PROTEIN EXTRACTION

The gastrocnemius muscles were removed, washed with ice cold PBS1X and frozen in liquid nitrogen. Frozen myocytes and skeletal muscle were transferred into lysis buffer (50 mM TRIS pH 7.5, 150 mM NaCl, 1 mM EDTA, 1 mM PMSF, and Complete^®^ protease inhibitor cocktail from Roche). Tissues were then homogenized using a Polytron. Triton Tx-100 was added to a final concentration of 1% and solubilization occurred by rotating for 1 h at 4°C. The soluble fraction obtained after 15 min of centrifugation at 13,000 *g* (4°C) was used for the experiments. In order to load each lane of the SDS-PAGE with equivalent amounts of total protein, the protein concentration of each lysate was measured in triplicate by Bradford assay using a BSA standard curve.

### WESTERN BLOTS

The western blotting conditions have been previously described (Gavillet et al., 2006). The polyclonal dystrophin antibody directed against the protein N-terminus (Dys12) was provided by M. Schaub (University of Zurich). The monoclonal dystrophin antibody (MANDYS8) and polyclonal actin antibody (A2066) were obtained from SIGMA. The polyclonal Na_v_1.5 antibody (ASC-005) was purchased from Alomone (Jerusalem, Israel).

### MICE VENTRICULAR MYOCYTE mRNA EXTRACTION

mRNA was extracted from frozen myocytes using the RNeasy Mini Kit, according to the manufacturer’s protocol (Qiagen, Hombrechtikon, Switzerland). cDNA was synthesized from 1 μg of RNA using the MU-MLV reverse transcriptase, according to the manufacturer’s protocol (Q-Biogene EMMLV100, Irvine, USA). Fifty nanograms of cDNA combined with 1x TaqMan Universal Master Mix (Applied Biosystems, Foster, USA) and 1 μl of probe were loaded into each well. The *SCN5A* probe (Mm00451971), the glyceraldehyde-3-phosphate dehydrogenase (GAPDH) probe (Mm99999915), the *SCN1B* probe (Mm00441210) and the Nedd4-2 probe (Mm00459584) were obtained from Applied Biosystems. The 96 well thermal plate was cycled at 50°C for 2 min and 95°C for 10 min, followed by 40 cycles at 95°C for 15 s and 60°C for 1 min. GAPDH was used as a reference gene to normalize the data. The comparative threshold cycle relative quantification method was used to compare the amounts of mRNA in control and mdx^*5cv*^ mice. Samples were measured in duplicate.

### PATCH CLAMP EXPERIMENTS

Only rod-shaped myocytes with distinct edges were selected for patch clamp experiments. The whole-cell configuration of the patch-clamp technique was used to record *I*_Na_. Experiments were performed at room temperature (22–23°C). Current recordings were performed using a VE-2 (Alembic Instruments) amplifier. Borosilicate glass pipettes (tip resistance 1–2 MΩ) were filled with a solution containing 60 mM CsCl, 70 mM cesium aspartate, 1 mM CaCl_2_, 1 mM MgCl_2_, 10 mM HEPES (4-(2-hydroxyethyl)-1-piperazineethanesulfonic acid), 11 mM EGTA (ethylene glycol tetraacetic acid), and 5 mM Na_2_ATP (pH adjusted to 7.2 with CsOH). Myocytes were bathed with a solution containing 10 mM NaCl, 120 mM NMDG-Cl (*N*-methyl-D-glucamine chloride), 2 mM CaCl_2_, 1.2 mM MgCl_2_, 5 mM CsCl, 10 mM HEPES, and 5 mM glucose (pH adjusted to 7.4 with CsOH). Holding potentials were -120 mV and current densities (pA/pF) were obtained by dividing the peak *I*_Na_ by the cell capacitance obtained using the transient capacitive current caused by a +5 mV pulse from the holding potential. Peak currents were measured during a current voltage protocol. To quantify the voltage-dependence of steady-state activation and inactivation, data from individual cells were fitted with the Boltzmann relationship, *y*(*V*_m_) = 1/1 + exp[(*V*_m_ - *V*_1/2_)/*k*], in which y is the normalized current or conductance, *V*_1/2_ is the voltage at which half of the available channels are inactivated, *k* is the slope factor, and *V*_m_ is the membrane potential.

### STATISTICAL ANALYSES

Data were represented as mean values ± SEM. Two-tailed Student’s *t*-test was used to compare means. Statistical significance was set at *P* < 0.05.

## RESULTS

### THE PROTEASOME INHIBITOR MG132 RESCUES Na_v_1.5 PROTEIN LEVELS AND THE SODIUM CURRENT IN mdx ^*5cv*^ MICE

**FIGURE 1:**
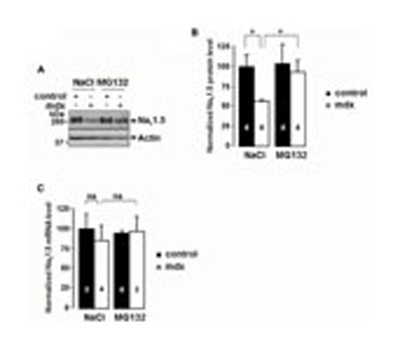
**Effects of MG132 treatment on Na_v_1.5 protein content and mRNA level.** **(A)** Representative Western blot of ventricular myocyte lysates of control and mdx^*5cv*^ mice treated with MG132 or 0.9% NaCl as indicated. Eighty micrograms of lysate were loaded in each lane. **(B)** Bar graph representing the amounts of total Na_v_1.5 protein in control and mdx^*5cv*^ ventricular myocytes quantified by digital density measurements. **(C)** Quantitative real time PCR experiments. Bar graph representing the amounts of *Scn5a* mRNA in control and mdx^*5cv*^ ventricular myocytes, analyzed by real time PCR (Taqman^®^), as described in the Material and Methods. The number of mice used for quantification is indicated in the bars. *P < 0.05.

The cardiac voltage-gated sodium channel, Na_v_1.5, is part of the DMC in mouse cardiomyocytes (Gavillet et al., 2006). The Na_v_1.5 protein content and the *I*_Na_ were both decreased in mdx^*5cv*^ mice, in which dystrophin is not expressed (Gavillet et al., 2006). In addition, it was shown that the sodium channel could be ubiquitylated by ubiquitin protein ligases of the Nedd4 family, thereby regulating the density of the channel at the cell membrane (van Bemmelen et al., 2004). In order to determine whether the ubiquitin proteasome system is implicated in the diminution of the sodium channel in the cardiomyocytes of dystrophin-deficient mice, control and mdx^*5cv*^ mice were treated with the proteasome inhibitor MG132. Osmotic mini pumps were implanted subcutaneously and delivered MG132 at a dose of 10 μg/kg/24 h over a 7-day period. Western blot experiments were performed using cardiomyocyte lysates of mdx^*5cv*^ and control mice, both treated with either MG132 or saline solution (0.9% NaCl). The protein content of Na_v_1.5 in the cardiomyocytes was quantified by digital density measurements of several Western blots, such as the one represented in **Figure 1A**. The total amount of Na_v_1.5 protein was decreased by 49 ± 3% in the ventricular myocytes of mdx^*5cv*^ mice treated with the saline solution, as compared to control mice (**Figures 1A,B**). The MG132 treatment increased the protein level of Na_v_1.5 in mdx^*5cv*^ cardiomyocytes to a level similar to that in control mice (**Figures 1A,B**). The proteasome inhibitor had no effect on the Na_v_1.5 protein content in control mice (**Figures 1A,B**). Finally, Na_v_1.5 mRNA quantification was performed using real time quantitative PCR. No significant difference of the Na_v_1.5 transcript between mdx^*5cv*^ and control mice was observed in either treatment (**Figure 1C**).

**FIGURE 2:**
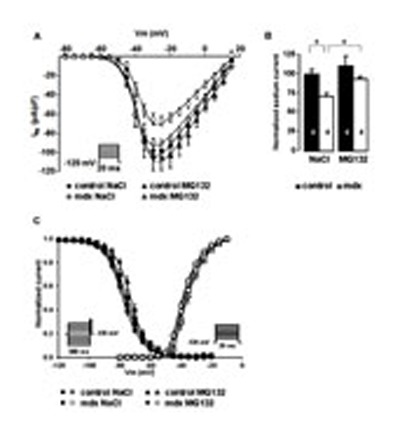
**Effects of MG132 treatment on the sodium current properties and mRNA level.** **(A)** Current density-voltage relationship of *I*_Na_ in control and mdx^*5cv*^ mice treated with MG132 or 0.9% NaCl, as indicated. The protocol is indicated in inset. **(B)** Bar graph quantifying the amounts of sodium current in control and mdx^*5cv*^ ventricular myocytes. Four cells were patched for each mouse and the number of mice used for quantification is indicated in the bars. The “normalized current” represents the maximum current density recorded at a given voltage (-25 mV). **(C)** Steady-state activation and inactivation curves. The protocol is indicated in inset. The number of mice used for quantification is indicated in the bars. Results are expressed as normalized mean signal intensity. *P < 0.05, n.s. not significant.

The *I*_Na_ was decreased by 29 ± 6% in mdx^*5cv*^ mice, as compared to that in the controls (**Figures 2A,B**). The proteasome inhibitor had a strong effect on the *I*_Na_ of mdx^*5cv*^ cardiac cells, increasing the current to a level similar to that found in control mice (**Figures 2A,B**). The effect of MG132 treatment on *I*_Na_ was restricted to an increase in the current density, since neither the voltage-dependence of activation nor the steady-state of inactivation were affected by the treatment (**Figure 2C**).

### MG132 TREATMENT DOES NOT RESCUE DYSTROPHIN EXPRESSION IN SKELETAL OR CARDIAC MUSCLES

**FIGURE 3:**
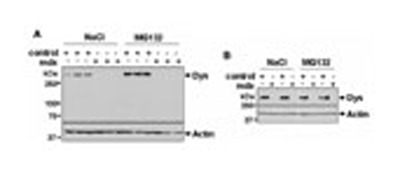
**Dystrophin is not expressed in skeletal muscle and in cardiomyocytes of mdx^*5cv*^ mice treated with MG132.** Western blots of mouse ventricular myocytes **(A)** and gastrocnemius muscle **(B)** lysates of control and mdx^*5cv*^ mice treated with MG132 or 0.9% NaCl, as indicated.

Bonuccelli et al. (2003) previously reported that the systemic treatment with 10 μg/Kg/24 h of MG132 rescued the expression of the dystrophin protein in skeletal muscle of the “original” mdx mouse strain. In the present study, Western blots of mdx^*5cv*^ gastrocnemial muscle lysates were performed in order to determine whether dystrophin is expressed in skeletal muscle upon treatment with MG132. The dystrophin antibody used for the Western blots was directed against the actin binding site in the N-terminus. The mdx^*5cv*^ mouse strain has a mutation in exon 10, which leads to a premature stop codon in the full-length transcript (Im et al., 1996). One can assume that if a shorter dystrophin form had been produced in mdx^*5cv*^ muscles upon MG132 treatment, it may have been detected. As expected, dystrophin expression was undetectable in cardiac and skeletal muscle lysates of mdx^*5cv*^ mice treated with 0.9% NaCl (**Figures 3A,B**). However, contrary to that described with the “original” mdx mice, MG132 treatment did not rescue the dystrophin expression in mdx^*5cv*^ skeletal muscle or cardiomyocytes (**Figures 3A,B**).

### Nedd4-2 AND THE β1-SUBUNIT mRNA AMOUNTS ARE NOT MODIFIED BY MG132 TREATMENT

**FIGURE 4:**
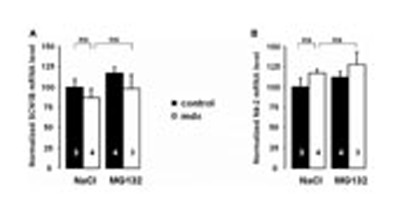
**MG132 treatment does not modify the mRNA expression level of *SCN1B* and Nedd4-2 genes.** Bar graph representing the amounts of *SCN1B* **(A)** and Nedd4-2 **(B)** mRNA in control and mdx^*5cv*^ ventricular myocytes, analyzed by quantitative real time PCR (Taqman^®^) as described in the Material and Methods. The number of mice used for quantification is indicated in the bars. Results are expressed as normalized mean signal intensity. n.s. not significant.

Na_v_1.5 was shown to be regulated by the ubiquitin ligase protein Nedd4-2, which is expressed in the heart (van Bemmelen et al., 2004; Rougier et al., 2005). The β-subunits of Na_v_1.5 were shown to modulate channel activity (Yu et al., 2005). In addition, the β1-subunit of Na_v_1.5 (encoded by the gene *SCN1B*) was described to be down-regulated in the skeletal muscle of DMD patients (Haslett et al., 2002). In order to determine whether these proteins play a role in the regulation of Na_v_1.5 in mdx^*5cv*^ mice treated with MG132 or 0.9% NaCl, real time quantitative PCR experiments were performed to quantify the relative amounts of mRNA. **Figures 4A,B** illustrate that there are no differences between the different tested conditions, suggesting that these proteins are not likely involved in the modulation of Na_v_1.5 upon MG132 treatment.

## DISCUSSION

Treatment of “original” dystrophin-deficient mice with the proteasome inhibitor MG132 was shown to rescue dystrophin expression in their skeletal muscle (Bonuccelli et al., 2003). The authors did not, however, investigate the effect of MG132 on cardiac muscle (Bonuccelli et al., 2003). In the mdx^*5cv*^ mouse strain, the Na_v_1.5 protein content is decreased by ~50% and the *I*_Na_ by ~30% (Gavillet et al., 2006). Studies using heterologous expression systems have demonstrated that ubiquitylation of Na_v_1.5 could trigger its internalization and decrease *I*_Na_ (van Bemmelen et al., 2004). In the present work, control and mdx^*5cv*^ mice were treated with MG132 in order to investigate the implications of the ubiquitin proteasome system on the regulation of Na_v_1.5 in cardiac cells. The main findings of this study are: (1) the proteasome inhibitor MG132 rescues the sodium channel Na_v_1.5 and *I*_Na_ in mdx^*5cv*^ cardiomyocytes, and (2) MG132 does not rescue the dystrophin expression in either cardiac or skeletal muscle in mdx^*5cv*^ mice.

The proteasome is a proteolytic complex which rapidly degrades ubiquitylated proteins (Rock et al., 1994). MG132 is a molecule which reversibly blocks protein degradation by the proteasome (Rock et al., 1994). The results of the present work suggest that the decrease of Na_v_1.5 observed in mdx^*5cv*^ mice could be either directly or indirectly mediated by the proteasome. It is more likely that the proteasome is indirectly implicated in the regulation of Na_v_1.5 since membrane proteins are primarily degraded by the lysosomal apparatus in eukaryotic cells, whereas the proteasome is involved in the proteolysis of cytosolic proteins (Lee and Goldberg, 1998). The activity of endocytic proteins is regulated by ubiquitin signals and the proteasome could control the degradation of these ubiquitylated proteins (Longva et al., 2002). Components of the endocytic machinery that undergo ubiquitylation are, however, primarily monoubiquitylated and the proteasome recognizes polyubiquitylated proteins. It has been suggested that endocytic proteins might be transiently polyubiquitylated and degraded by the proteasome (Salghetti et al., 2001). Altogether, these results suggest that the proteasome indirectly regulates Na_v_1.5. Additional experiments using endocytosis or lysosome inhibitors should be carried out to help identify the proteolytic pathways involved in the degradation of Na_v_1.5.

Unlike Bonuccelli et al. (2003), this study did not use the “original” mdx mouse strain which carries a premature stop codon in exon 23, since this strain was shown to have revertant fibers due to exon skipping events (Danko et al., 1992). This study used the mdx^*5cv*^ mouse strain which carries an A to T mutation in the middle of exon 10 that produces a new splice donor site and generates a premature stop codon in full-length transcripts (Im et al., 1996). MG132 treatment of mdx^*5cv*^ mice did not rescue dystrophin expression in skeletal or cardiac muscle. The different effects of MG132 treatment on the two mouse strains could be due to the nature of the dystrophin mutations. The mutation on the dystrophin gene of mdx^*5cv*^ mice may produce an unstable transcript which is not translated, whereas the “original” mdx strain may produce an unstable protein that accumulates upon MG132 treatment. This interpretation is supported by the study of Assereto et al. (2006) on the DMC composition of DMD and BMD muscle explants following *in vitro* treatment with 20 μM MG132. Only some of the DMD and BMD explants showed signs of DMC rescue after MG132 treatment, probably due to the nature of the dystrophin mutations.

In conclusion, it was observed that the proteasome inhibitor MG132 rescued the total amount of Na_v_1.5 protein and the *I*_Na_ in cardiomyocytes, but did not rescue dystrophin expression in dystrophin-deficient mdx^*5cv*^ mice. Moreover these results suggest that the proteasomal pathway is implicated in the degradation of Na_v_1.5 channel in dystrophinopathies. We have yet to determine if the proteasome is directly or indirectly involved in the degradation of polyubiquitylated Na_v_1.5 channel or if it regulates the endocytic machinery which controls the density of the sodium channel at the plasma membrane. Additional experiments on the mechanisms of Na_v_1.5 channel degradation and regulation in WT and dystrophin-deficient cardiac cells are needed to better understand the pathways involved in the maintenance of the Na_v_1.5 channel in specific pools.

## Conflict of Interest Statement

The authors declare that the research was conducted in the absence of any commercial or financial relationships that could be construed as a potential conflict of interest.
